# Are brain MRI abnormalities associated with the semiology of functional seizures?

**DOI:** 10.1002/brb3.2882

**Published:** 2023-01-09

**Authors:** Ali A. Asadi‐Pooya, Wesley T. Kerr, Ioannis Karakis, Kousuke Kanemoto, Anilu Daza‐Restrepo, Mohsen Farazdaghi, Faith J. Horbatch, Nicholas J. Beimer, Dawn E. Eliashiv, Aida Risman, Yuko Sugimoto, Brenda Giagante

**Affiliations:** ^1^ Epilepsy Research Center Shiraz University of Medical Sciences Shiraz Iran; ^2^ Jefferson Comprehensive Epilepsy Center, Department of Neurology Thomas Jefferson University Philadelphia Pennsylvania; ^3^ Department of Neurology University of Michigan Ann Arbor Michigan; ^4^ Department of Neurology David Geffen School of Medicine at the University of California Los Angeles California; ^5^ Department of Neurology Emory University School of Medicine Atlanta Georgia; ^6^ Neuropsychiatric Department Aichi Medical University Nagakute Aichi Japan; ^7^ ENyS CONICET – Neuroscience and Epilepsy Service El Cruce Hospital “Dr. Néstor Kirchner,” Buenos Aires Argentina

**Keywords:** magnetic resonance imaging (MRI), neuropsychiatrics, psychiatric disorders

## Abstract

**Purpose:**

To investigate whether radiologically apparent brain magnetic resonance imaging (MRI) abnormalities are associated with the functional seizure (FS) semiology.

**Methods:**

All patients with a diagnosis of FS at the epilepsy centers at Shiraz University of Medical Sciences, Iran; Aichi Medical University Hospital, Japan; University of Michigan, USA; University of California, Los Angeles, USA; Emory University School of Medicine, USA; and Hospital el Cruce, Argentina, were studied.

**Results:**

One hundred patients were included; 77 (77%) had motor functional seizures. Lobar location of brain abnormality did not have an association with the semiology (*p* = .83). There was no significant difference between ictal behaviors in patients with frontal or parietal lesions compared to those with temporal or occipital lesions.

**Conclusion:**

There were no associations between functional seizure ictal behaviors and locations of the radiologically apparent brain MRI abnormalities. Further studies are needed to evaluate the underpinnings of varying behaviors in FS.

## 1 INTRODUCTION

Functional seizures (FS) comprise as much as 10% of patients seen at comprehensive epilepsy centers; they happen in a heterogeneous patient population and their underlying etiology is not fully clear yet (Asadi‐Pooya, 2021,; 2017; Popkirov et al., 2019). While each patient's ictal behavior can be unique, clustering analyses have demonstrated key behavioral subtypes including motor functional seizures and akinetic functional seizures (Asadi‐Pooya & Farazdaghi, 2022).

Abnormal brain magnetic resonance imaging (MRI) in patients with FS have often been dismissed as incidental findings; however, there is increasing evidence that FS are associated with both structural brain abnormalities and functional brain connectivity abnormalities (Asadi‐Pooya & Homayoun, 2020; Foroughi et al., 2020; Kerr et al., 2021; McSweeney et al., 2017; Tavakoli Yaraki et al., 2022). These abnormalities can be associated with some recognized factors associated with FS, including traumatic brain injury (Asadi‐Pooya & Farazdaghi, 2021). Broadly, these neuroimaging abnormalities may reflect changes in the connectivity between limbic and affective areas, frontal executive control, and the motor networks (Foroughi et al., 2020).

The aim of the current study was to investigate whether radiologically apparent brain MRI abnormalities are associated with the functional seizure semiology. Just as different symptomatogenic zones in epileptic seizures produce varying ictal behaviors, we hypothesized that varying neuroimaging abnormalities may be associated with varying ictal behaviors in FS. In specific, we hypothesized that the frontal and parietal lesions are associated with motor semiology while temporal and occipital lesions are associated with nonmotor (akinetic) semiology.

## 2 METHODS

### 2.1 Participants

All patients (16–70 years of age) with a documented electroclinical diagnosis of FS, based on the International League Against Epilepsy certainty criteria (LaFrance et al., 2013), who were diagnosed at the epilepsy centers at Shiraz University of Medical Sciences, Iran; Aichi Medical University Hospital, Japan; University of Michigan, USA; University of California, Los Angeles, USA; Emory University School of Medicine, USA; and Hospital el Cruce, Argentina, were studied. The time periods for data collections were different between the centers and ranged from 2008 to 2021. Patients with comorbid epilepsy were excluded. Because our hypothesis relied on patterns in brain MRI abnormalities, patients with normal brain MRIs or without such neuroimaging were excluded (we did not keep track of such patients).

### 2.2 Data collection

Age at functional seizure onset, age at diagnosis, sex, seizure semiology (motor functional seizures versus akinetic functional seizures), brain MRI findings (nature of the finding [e.g., nonspecific white matter changes, atrophy, gliosis, tumor, etc.], and the lobar location of the pathology by visual inspection [by a radiologist and a neurologist] [right or left and the involved lobe(s)]), and the associated factors (a history of physical abuse, sexual abuse, family dysfunction, any medical comorbidities) were registered. We classified ictal behaviors into motor and akinetic subtypes based on the observed behavior during the video‐EEG monitoring and not based on the patient's and caregiver's descriptions. If a patient had multiple seizure types during their video‐EEG monitoring, including motor seizures, we arbitrarily classified them as having motor functional seizures for the statistical analyses (due to the small sample size and in order to make the statistical analyses doable). Brain MRIs at all centers included epilepsy protocol, 1.5 Tesla (or above) neuroimaging; however, this was done according to each center's routine protocols (details may vary between the centers).

### 2.3 Statistical analyses

Values were presented as number (percent) of subjects for categorical variables and as mean ± standard deviation for continuous variables. The IBM SPSS Statistics (version 25.0) was used for the statistical analyses. Pearson's chi‐square test was used. A *p* value (2‐sided) less than .05 was considered as significant.

### 2.4 Standard protocol approvals, registrations, and patient consents

The Institutional Review Boards of all the centers approved this study.

## 3 RESULTS

In total, 100 patients were included (female: 64 [64%]). Seventy‐seven patients (77%) had motor functional seizures and 23 patients (23%) had akinetic functional seizures (24 patients had both seizure types and were classified as having motor functional seizures). Table 1 shows the demographic and clinical characteristics of the patients. Lobar location of the brain MRI abnormality did not have a significant association with the FS semiology (*p* = .83 for all lobes). There was no significant difference (*p* = .37) between ictal behavior subtypes in patients with frontal (*N* = 28) or parietal (*N* = 3) lesions (motor FS in 22) compared with those who had temporal (*N* = 20) or occipital (*N* = 2) lesions (motor FS in 16). There was no significant difference (*p* = .82) in ictal behaviors between patients with right‐sided (*N* = 21, motor FS in 17), left‐sided (*N* = 28, motor FS in 22), or those with bilateral lesions (*N* = 51, motor FS in 38) either. The specific neuroimaging findings were very diverse and there were no consistent patterns amenable to statistical analysis. Furthermore, it seems that the types of brain MRI abnormalities seen (Table 1) may align more with the patients’ medical comorbidities (e.g., 18 patients had chronic headache that can cause white matter changes and 10 people had hypertension that can cause vascular lesions).

**TABLE 1:** Demographic and clinical characteristics of the patients

Sex (female: male)	64: 33 (3 missing)
Age at onset (years)	Mean: 30; median: 28; standard deviation: 14
Age at diagnosis (years)	Mean: 38; median: 36; standard deviation: 14
A history of physical abuse	16 (16%)
A history of sexual abuse	10 (10%)
A history of family dysfunction	26 (26%)
A family history of seizures	23 (23%)
Medical comorbidities	54 (54%) (chronic headache in 18, hypertension in 10, thyroid problems in 8, diabetes in 5, etc.)
Brain MRI abnormality	White matter changes: 38; vascular: 11; gliosis: 8; cyst: 5; others (anomalies, tumors, etc.): 38
Brain MRI abnormality location	Frontal: 28; temporal: 20; parieto‐occipital: 5; multiple: 47
Brain MRI abnormality location	Right: 21; left: 28; bilateral: 51

MRI, magnetic resonance imaging.

## 4 DISCUSSION

In the current study, we did not observe a significant association between functional seizure motor semiology and neuroimaging abnormality location (i.e., lobe or side of the lesion). In one previous study of 206 patients with FS, markers of brain abnormalities (e.g., epileptiform EEG changes, MRI abnormalities, and neuropsychological deficits) were studied to explore whether brain disorders were associated with an increased risk of FS (Reuber et al., 2002). At least one marker of brain disorder was detected in 22% of the patients (MRI changes in 27% of those examined or 9.7% of the whole group, epileptiform potentials in 8.7%, and neuropsychological deficits in 9.7% of the whole group). The authors concluded that brain abnormalities play a role in the development of FS (Reuber et al., 2002). Furthermore, there is increasing evidence that FS are associated with structural and functional brain abnormalities (Asadi‐Pooya & Homayoun, 2020; Foroughi et al., 2020; Kerr et al., 2021; McSweeney et al., 2017; Tavakoli Yaraki et al., 2022). Therefore, it may be worthwhile to pursue the hypothesis of “varying neuroimaging abnormalities may be associated with varying ictal behaviors in FS” by using advanced neuroimaging techniques (see below).

In our study, the nature of neuroimaging abnormalities was diverse and there were no clear patterns in the observed abnormalities even within lobar locations. This marked diversity was also observed in the results of studies of radiologically apparent abnormalities and quantitative structural and functional neuroimaging associations with FS (McSweeney et al., 2017). This indicates that there may be other important factors that contribute to ictal behaviors in FS that were not apparent in the radiological analysis of brain MRIs.

During the past two decades, many investigators have tried to study brain abnormalities in patients with FS. These studies have applied various techniques including, functional magnetic resonance imaging (fMRI), EEG, MRI with or without diffusion tensor imaging (DTI), and magnetoencephalography (MEG), among others (Foroughi et al., 2020). These studies have identified a variety of brain connectivity (functional and structural) abnormalities in patients with FS; the most consistent findings included connectivity abnormalities between brain regions such as sensorimotor cortex, frontal lobes, limbic system, temporoparietal junction, basal ganglia, occipital lobes, and uncinate fasciculus. However, none of these studies provided a high level of evidence (all of the studies were either cross sectional or retrospective studies with limited sample sizes and most of the studies did not match their cases and their controls with respect to their psychiatric comorbidities) (Foroughi et al., 2020).

It seems that pursuing the concept of brain abnormalities (structural and functional) in patients with FS may result in a breakthrough in identifying the neurobiological underpinnings of FS; however, well‐designed large multicenter studies are needed to investigate this concept.

## 5 LIMITATIONS

This study focused on radiologically apparent abnormalities on clinically obtained MRIs. While a significant minority of patients with FS have neuroimaging abnormalities, this still reflects a minority of patients. To address this concern, we combined results from six international Comprehensive Epilepsy Centers to create a relatively large sample size. However, the underlying heterogeneity of abnormalities and factors associated with FS remained broad, indicating that further multisite collaborations are necessary (Perez et al., 2021). Additionally, we focused on radiologically apparent abnormalities. Functional neurological disorders (e.g., FS) are defined by the presence of distressing neurological symptoms that are not explained by readily identifiable structural or physiological pathological changes capable of explaining the clinical presentation. Advanced neuroimaging postprocessing techniques and quantitative analysis of MRI morphology may reveal associations that are not appreciated by visual analysis. Finally, we did not have a control group in the current study.

## 6 CONCLUSION

There were no clear associations between functional seizure ictal behaviors and locations of the radiologically apparent brain MRI abnormalities. Further studies are needed to evaluate the biological underpinnings of varying ictal behaviors in patients with FS.

## AUTHOR CONTRIBUTIONS

Ali A. Asadi‐Pooya: study design, data collection, statistical analyses, and manuscript preparation. Others: data collection and manuscript preparation.

## CONFLICT OF INTEREST

Ali A. Asadi‐Pooya: Honoraria from Cobel Daruo, Tekaje, Sanofi, and RaymandRad; Royalty: Oxford University Press (Book publication); Grant from the National Institute for Medical Research Development. Wesley T. Kerr received honoraria from Medlink Neurology for articles on this topic and personal compensation for consulting with SK Lifesciences. Others: no conflict of interest.

### PEER REVIEW

The peer review history for this article is available at https://publons.com/publon/10.1002/brb3.2882.

## Data Availability

The data are confidential and will not be shared.
